# Supplementary material for: Multimodal AI and tumour microenvironment integration predicts metastasis in cutaneous melanoma
Source: Nat Commun. 2025 Nov 18;16:10095. doi: 10.1038/s41467-025-65051-0 (PMC12627701; doi:10.1038/s41467-025-65051-0)
Supplement: Supplementary file 1 — Supplementary Information [file 41467_2025_65051_MOESM1_ESM.pdf]

Supplementary Information

Supplementary Table 1							
**Characteristics**	**Dataset, WSI No.**						**p-value**
	University Hospital North Durham, Durham, UK (n=527)	James Cook University Hospital, Middlesborough, UK (n= 707)	Roswell Park Comprehensive Cancer Center, Buffalo, USA (n= 693)	Hospital Clinic Barcelona, Barcelona, Spain (n=375)	Peter MacCallum Cancer Centre, Melbourne, Australia (n=878)	The Northern Ireland Tissue Biobank, Belfast, UK (n= 477)	
**Age at diagnosis, median (range), y**	52 (20-90)	56 (18-94)	61 (18-92)	56(18-90)	57 (18-92)	62 (20-98)	.056 (ANOVA)
**Sex (%)**							
Female	59.2	61.3	55.7	52.1	54.7	52.8	.75 (Chi^2)
Male	40.8	38.7	44.3	47.9	45.3	47.2	
**Breslow Depth, median (range), mm**	0.7 (0.10-2.0)	0.8 (0.12-8.0)	0.8 (0.1-2.0)	1.52 (0.9-5.5)	1.0 (0.18-8.0)	1.3 (0.1-11.7)	0.0002 (ANOVA)
**Miotic Count, median (range), mitoses/mm^2**	0.5 (0-18)	0.5 (0-13)	0.5 (0-35)	2.0 (0-32)	1.0 (0-21)	0.5 (0-21)	.039 (ANOVA)
**Anatomical Site (%)**							
Head and Neck	40.5	41.7	27.3	32.6	28.8	24.5	.086 (Chi^2)
Upper Limb	14.8	16.0	24.1	16.3	30.9	25.2	
Lower Limb	16.7	17.9	18.9	23.9	20.5	18.9	
Trunk	28.1	29.3	32.2	47.6	37.1	31.4	
**Histological Subtype (%)**							
Superficial Spreading Melanoma	70.7	71.9	72.2	79.3	79.8	74.9	.22 (Chi^2)
Nodular Melanoma	20.2	21.4	20.2	15.9	14.5	25.1	
Lentigo Maligna Melanoma	7.9	9.1	7.6	6.0	7.5	0	
**Metastasis (%)**							
Present	8.7	10.4	9.1	12.7	11.9	16.4	.56 (Chi^2)
Absent	91.3	89.6	90.9	87.3	88.1	83.6	
**Time to Metastasis, median (range), m**	86.5 (2-167)	86 (1-238)	69 (2-245)	94 (3-197)	67 (1-142)	87 (3-234)	.017 (ANOVA)
All p-values were derived using two-sided tests (Chi-squared for categorical variables, one-way ANOVA for continuous variables							

Supplementary Table 2: Comparison of Time-Dependent Brier Score Performance of Model/Feature Selection

	Time Point (months)	Cox	RSF	DeepSurv
**Staging Features Only**	12	0.14	0.15	0.12
	24	0.11	0.12	0.10
	60	0.09	0.10	0.09
**Clinical Features Only**	12	0.11	0.12	0.1
	24	0.09	0.09	0.08
	60	0.07	0.07	0.07
**Imaging Features Only**	12	0.13	0.16	0.11
	24	0.11	0.13	0.10
	60	0.09	0.11	0.09
**Multimodal (Combined Clinical and Imaging Features)**	12	0.11	0.11*	0.1
	24	0.09	0.09*	0.08
	60	0.07	0.06*	0.07

Cox - Cox Proportionate Hazard Survival Analysis
RSF – Random Survival Forest
***Melanoma Multimodal AI Prognostication (MelanoMAP)**

Supplementary Table 3. Features Excluded Due to Collinearity

Excluded Feature Due to Collinearity	Correlated Included Feature
Max Lost Expression	Loss of Microenvironmental Signal
Mean Lost Expression Prob_lo	
Min Lost Expression Prob_lo	
Max Maintained Expression Prob_lo	Mean Microenvironmental Signal
Min Maintained Expression Prob_lo	
Percentage Maintained Expression_lo	
Std Lost Expression Prob_lo	Microenvironmental Intensity Variability
Std Maintained Expression Prob_lo	
Total Patches_lo	Number of Microenvironmental Regions
am_pixels_am	Mean Area of Microenvironmental Regions
am_mean_am	Mean Microenvironmental Intensity
intensity_kurtosis_am	
intensity_median_am	
intensity_q25_am	Microenvironmental Intensity Skewness
intensity_q75_am	
morph_mean_eccentricity_am	Mean Compactness of Microenvironment
morph_mean_solidity_am	
morph_total_stained_area_am	Mean Area of Microenvironmental Regions
spatial_clustering_am	Spatial Density
spatial_mean_x_am	
spatial_mean_y_am	
spatial_std_x_am	Spatial Spread
spatial_std_y_am	
texture_correlation_am	Textural Homogeneity
texture_dissimilarity_am	

Collinearity checks excluded features with correlations exceeding ±0.7

Supplementary Table 4. List of Python Packages	
**Package**	**Version**
Pandas	2.2.2
Numpy	1.26.4
Shap	0.45.1
Tensorflow	2.16.1
Keras	3.3.3
Lifelines	0.28.0
Scikit-learn (sklearn)	1.5.0
Matplotlib	3.9.0
Scikit-Survival	0.22.2
PyTorch	2.3.0
OpenCV	4.x
SciPy	1.11.3
Seaborn	0.13.0
Statsmodels	0.14.0
torchvision	0.16.0
Numba	0.58.1
tqdm	4.66.0

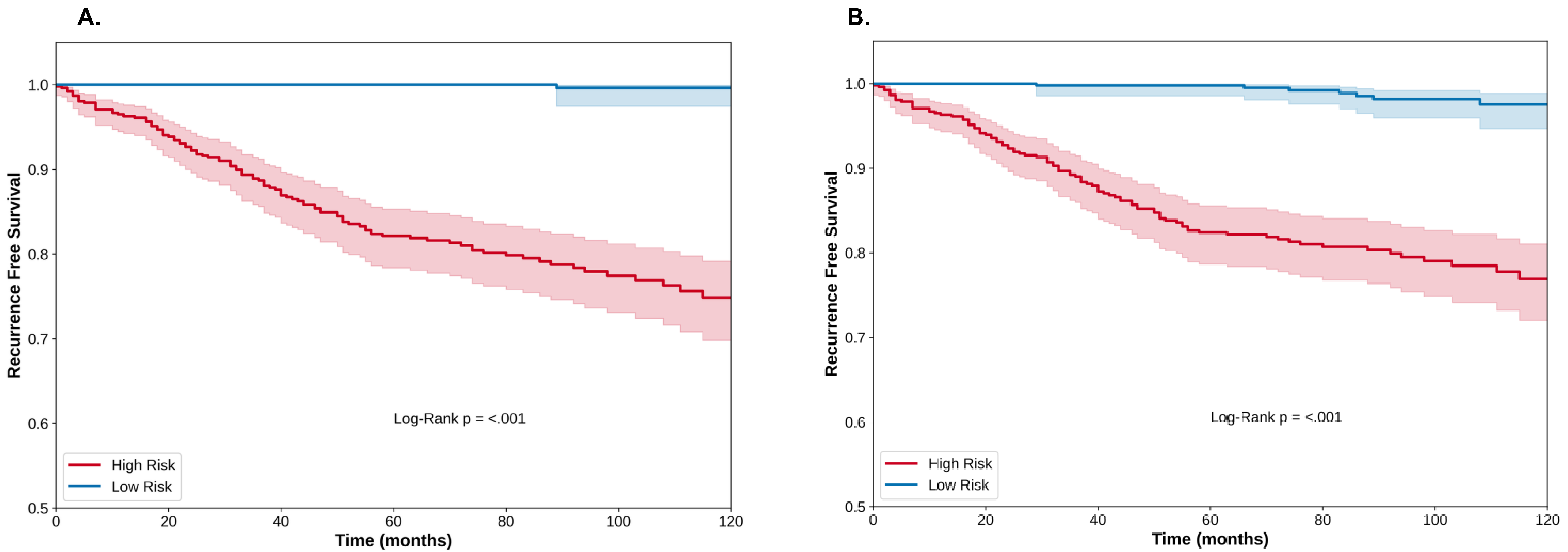

Supplementary Figure 1: Kaplan-Meier survival analysis comparing MelanoMAP and clinicopathological models in stratifying patients into high-risk and low-risk groups. A. Recurrence-free survival curves generated by the MelanoMAP model in the test cohort, incorporating data from the multinational database. **B.** Survival curves from the clinicopathological model applied to the same cohort. The center line indicates the Kaplan-Meier estimate of survival probability; shaded areas represent 95% confidence intervals. The log-rank test ($p < .001$) confirms a statistically significant difference in recurrence-free survival between risk groups.
